# Implementation of mRNA–Lipid Nanoparticle Technology in Atlantic Salmon (*Salmo salar*)

**DOI:** 10.3390/vaccines12070788

**Published:** 2024-07-18

**Authors:** Lars Ole Sti Dahl, Sjoerd Hak, Stine Braaen, Alicja Molska, Francesca Rodà, Jeremie Parot, Øystein Wessel, Johanna Hol Fosse, Håvard Bjørgen, Sven Even Borgos, Espen Rimstad

**Affiliations:** 1 Faculty of Veterinary Medicine, Norwegian University of Life Sciences, 1433 Ås, Norway; lars.ole.sti.dahl@nmbu.no (L.O.S.D.); stine.braaen@nmbu.no (S.B.); oystein.wessel@nmbu.no (Ø.W.); havard.bjorgen@nmbu.no (H.B.); 2 Department of Biotechnology and Nanomedicine, SINTEF Industry, 7034 Trondheim, Norway; sjoerd.hak@sintef.no (S.H.); alicja.molska@sintef.no (A.M.); froda@dongnocchi.it (F.R.); jeremie.parot@sintef.no (J.P.); sveneven.borgos@sintef.no (S.E.B.); 3 Clinical and Experimental Medicine PhD Program, University of Modena and Reggio Emilia, 41121 Modena, Italy; 4 IRCCS Fondazione Don Carlo Gnocchi ONLUS, 20148 Milan, Italy; 5 Norwegian Veterinary Institute, 1433 Ås, Norway; johanna.hol.fosse@vetinst.no

**Keywords:** mRNA vaccination, lipid nanoparticle technology, Atlantic salmon

## Abstract

Background: This study was conducted to investigate whether mRNA vaccine technology could be adapted for the ectothermic vertebrate Atlantic salmon (*Salmo salar*). Lipid nanoparticle (LNP) technology has been developed and optimized for mRNA vaccines in mammals, stabilizing mRNA and facilitating its delivery into cells. However, its utility at the temperatures and specific biological environments present in ectotherms remains unclear. In addition, it is unknown if modified mRNA containing non-canonical nucleotides can correctly translate in salmonid cells. Methods: We used an mRNA transcript coding for enhanced green fluorescence protein, flanked by the untranslated regions of the hemagglutinin-esterase gene of the infectious salmon anemia virus, and a 120-base-long poly(A) tail. The mRNA was generated via in vitro transcription where uridine residues were replaced with N1-methyl-pseudouridines, and then encapsulated in LNPs. Results: When transfected into the salmonid cell line CHH-1, the mRNA-LNP construct induced expression of EGFP. Furthermore, when mRNA-LNPs were injected intramuscularly into salmon, in vivo protein expression was demonstrated via immunohistochemistry. EGFP was observed in cells infiltrating the spaces between muscle cells in a focal inflammatory response. Conclusion: The results indicate that N1-methyl-pseudouridine-modified mRNA encapsulated in LNPs can be used to express antigens of interest in salmonid fish.

## 1. Introduction

Infectious diseases represent a major problem for the farming of salmonid fish. Attempts at immunization were made as early as the 1930s [1], and the first licensed vaccine, targeting enteric redmouth in rainbow trout (*Oncorhynchus mykiss*), was launched in 1976 [2]. Most commercial vaccines used in salmonid aquaculture today are inactivated and accompanied by an oil-based adjuvant that may cause adverse side effects [3,4,5]. The first commercial DNA vaccine in any farmed animal was against infectious hemorrhagic necrosis in Atlantic salmon (*Salmo salar*), a milestone that demonstrated that nucleic acid-based vaccines can induce protection in salmonid fish [6]. However, DNA vaccines have some limitations, such as low immunogenicity [7] and the theoretical possibility of DNA integration into the host genome lineage [8].

The success of mRNA vaccines against COVID-19 has demonstrated their potential in preventing infectious diseases [9,10]. However, mRNA is inherently unstable under natural conditions [11], and exogenous mRNA entering cells may be recognized by a range of cellular pattern recognition receptors, such as membrane-bound Toll-like receptors and cytosolic RIG-I-like receptors [12,13,14,15]. Consequently, the stabilization of the mRNA, protection against its degradation, and the prevention of adverse immune responses are prerequisites for achieving an efficient response to mRNA vaccines. The discovery that certain chemical modifications of endogenous nucleotides can reduce the innate immune response [16] led to the advancement of using pseudouridine in exogenous mRNA transcripts. This increased mRNA stability, reduced innate immune responses, and enhanced antigen translation [16,17]. Further enhancement of mRNA stability and translational capacity were achieved through poly(A) tail-length optimization, which has been found to be an important factor [18]. However, the inefficient transfer of exogenous mRNA into cells, due to the negative charge of mRNA, and the common presence of extracellular RNases has limited the medical use of naked mRNA [19,20].

Although the use of cationic polymers and lipids has been widely and successfully applied for mRNA studies in cell cultures, their positive charge causes systemic toxicity and has prohibited their use in live animals. It was not until the development of so-called ionizable lipids that RNA could be safely applied in vivo in synthetic carriers [21,22]. Ionizable lipids allow for efficient complexation with negatively charged RNA and subsequent LNP encapsulation during LNP assembly at pH 4. These lipids are neutral at physiological pH but regain their charge after entering cells and the intracellular acidic endosomal compartment; this re-charging is thought to promote RNA endosomal escape [23]. In addition to making it possible to achieve efficient antigen production after RNA injection in live animals, mRNA-LNPs have also been found to have an adjuvant effect [24,25]. Together, these advancements have made it possible to use mRNA for eliciting a strong immune response to the encoded antigen and thus to use it in vaccines [26]. Implementing RNA-based vaccines against viral diseases in aquaculture could be a useful prophylactic tool and possibly resolve some of the shortcomings of other types of vaccines.

Differences between mammalian and fish immune responses could influence the effect of mRNA vaccines in salmonid fish. The foreign mRNA needs to be transported into cells, which is facilitated by LNPs. LNPs were designed to evade detection by mammalian opsonins and innate immune cells. However, it has been suggested that piscine innate immune molecules have greater diversity than those in mammals [27,28,29]. This raises the question of whether the increased receptor diversity in piscine innate immune molecules is more likely to recognize and degrade mRNA-LNPs prior to cellular uptake. This could potentially result in a reduced circulation time of the mRNA-LNP molecules, consequently leading to a decreased antigen production.

Even though the mechanisms underlying the cellular uptake of LNPs, which is dependent on the administration route as well as the size and physicochemical properties of the LNPs, are not yet fully understood, the endocytosis pathway is proposed to be the predominant route for cellular entry of these particles [30]. Therefore, differences in cell membrane composition between mammals and fish may alter the efficiency of this uptake process. In vitro studies have indicated that the phospholipid composition and cholesterol contents of different fish cells are similar to those of mammalian cells, but the cells’ membrane tension differs [31]. Similarities in lipid composition between mammalian and salmon leukocytes have also been reported [32]. However, it remains unclear whether potential differences in the membrane receptors involved in receptor-mediated endocytosis pathways between mammals and fish affect the ability of piscine cells to internalize mRNA-LNPs.

Studies on the use of LNP-encapsulated mRNA have shown differences in the in vivo delivery of mRNA and cellular responses to LNPs [33], even between mammalian species. It thus seems likely that differences between mammals and fish exist. The scarce information about mRNA-based vaccines in fish underscores the importance of further research, particularly considering the essential role that the fish industry plays in the global economy and as a food source.

The aim of this study was to assess whether mRNA-LNP technology can induce protein expression in Atlantic salmon, and thus whether it could present a viable strategy for developing vaccines against salmonid pathogens. To do so, N1-methyl-pseudouridine-modified mRNAs encoding enhanced green fluorescence protein (EGFP) were synthesized through in vitro transcription (IVT) and subsequently encapsulated in LNPs. In vitro expression was evaluated through transfection of the mRNA-LNPs in a salmonid cell line, while in vivo expression was evaluated by intramuscular injection of the mRNA-LNPs in Atlantic salmon. EGFP was detected by fluorescence or immunohistochemical staining.

## 2. Materials and Methods

### 2.1. Design of DNA Plasmids

A plasmid (pVax1 Vector) was designed with an SP6 promoter and the coding sequence of the EGFP gene, flanked by 5′ and 3′ untranslated regions (UTRs) from the hemagglutinin-esterase (HE) gene of infectious salmon anemia virus (ISAV). Additionally, a 3′ poly(A) of 120 residues was added to the plasmid construct. MluI restriction sites were inserted upstream and downstream of the 5′ UTR and the poly(A) region, respectively. The plasmid encoded a 988-nucleotides (nt)-long mRNA construct and was synthesized using GenScript (Piscataway, NJ, USA). The sequence of the mRNA construct is given in Appendix A.

### 2.2. In Vitro Transcription of mRNA

**Figure 1:**
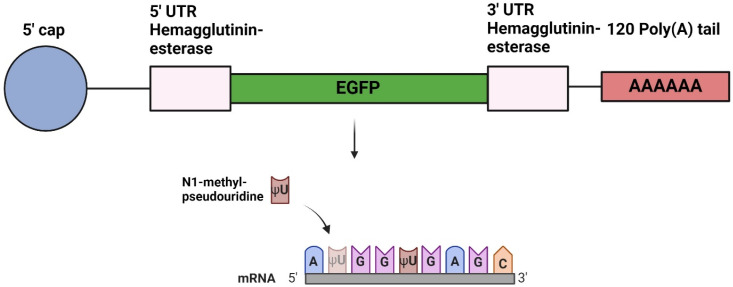
Overview of mRNA construct. The mRNA was generated from a plasmid encoding EGFP with untranslated regions from the hemagglutinin-esterase gene and a 120-residue poly(A) tail. In addition, uridine residues were substituted with N1-methyl-pseudouridines, and a 5′ cap was enzymatically added post-transcriptionally. Created with BioRender.com.

Plasmids were linearized by the MluI restriction enzyme and purified with the NucleoSpin Gel and PCR Clean-up kit (Macherey-Nagel, Düren, Germany). The IVT was carried out using the RiboMAX™ Large Scale RNA Production System–SP6 (Promega, Madison, WI, USA), following the manufacturer’s recommendations. Linearized DNA encoding Firefly luciferase (Fluc), which was included in the kit, was used as a control with an expected mRNA size of 1800 nt. Uridine-5′-triphosphates were replaced with N1-methyl-pseudouridine-5′-triphosphates (TriLink Biotechnologies, San Diego, CA, USA). The resulting IVT mRNA was purified using the RNeasy mini kit (QIAGEN, Hilden, Germany). Enzymatic capping of the mRNAs was performed post-transcriptionally with the ScriptCap™ Cap 1 Capping System (Cellscript Inc., Madison, WI, USA), and the nucleoside-modified, capped mRNAs were purified using the RNeasy mini kit (QIAGEN) (Figure 1). Finally, RNA quality and integrity were analyzed using capillary gel electrophoresis with the use of a Tapestation 4200 instrument (Agilent, Santa Clara, CA, USA) and an RNA ScreenTape ladder (Agilent). The resulting mRNA was stored at −80 °C until use.

### 2.3. mRNA-LNP Formulation

mRNA-LNPs were formulated using a flow-mixing-based approach. Stock solutions of ionizable lipid SM-102, cholesterol, DSPC, and PEG2000-DMG were prepared in EtOH. Lipids were mixed at a molar ratio (%) of 50:38.5:10:1.5, SM-102/cholesterol/DSPC/PEG2000-DMG, and the EtOH volume was adjusted to obtain a 6.18 mg/mL (10 mM) total lipid concentration. The mRNA was dissolved in RNase-free 25 mM acetate buffer (pH 4.0) at 39 μg/mL. The lipid mixture in EtOH and the mRNA solution in acetate buffer were placed in syringes on computer-operated syringe pumps and mixed in a T-junction mixer at a total flow rate of 12 mL/min and an EtOH–buffer flow rate ratio of 1:3, resulting in a 1:30 mRNA–ionizable lipid weight ratio. After formulation, the mRNA-LNPs were dialyzed using a 10 kDa molecular weight cut-off dialysis cassette (Slide-A-Lyzer™ G2 Dialysis Cassettes, Thermo Scientific™) overnight at room temperature against Tris buffer (10 mM, pH 7.4). After dialysis, the mRNA-LNPs’ solution was adjusted with 10% sucrose, the mRNA concentration was measured (RiboGreen assay; see below), and the mRNA-LNPs were diluted to an mRNA concentration of 10 ug/mL using Tris buffer (10 mM, pH 7.4) with 10 weight% sucrose. The mRNA-LNPs were stored at −20 °C in aliquots. On the day of the experiments, corresponding aliquots were thawed and used the same day.

### 2.4. mRNA-LNP Characterization

Dynamic light scattering (DLS, Cordouan–VascoKIN) was used to measure mRNA-LNP size (Z-avg) and size distribution (polydispersity index, PDI) after a 40× mRNA-LNP dilution in Tris buffer (10 mM, pH 7.4). Multi-detector field flow fractionation (MD-FFF, AF2000 Multiflow FFF (Postnova Analytics, Landsberg am Lech, Germany)) was used, equipped with a multi-angle light-scattering detector (MALS, PN3621) and a UV–Vis absorbance detector (PN3211). MALS was used to assess mRNA-LNP size (radius of gyration, Rg) and size distribution and recovery was determined using the UV absorbance obtained after a direct injection (a measure for the total mass present in the sample) and the UV absorbance from the fraction containing the mRNA-LNPs only. The system was operated according to a published methodology [34].

To assess mRNA encapsulation and concentration, we used the Quant-it RiboGreen assay (Invitrogen, R11490, Waltham, MA, USA). Calibration curves (at mRNA concentrations of 0–640 ng/mL) were prepared using the encapsulated mRNA. mRNA-LNPs were assayed at multiple dilutions and both in the presence of 0.5% Triton X (to measure the total mRNA concentration, as Triton X disrupts mRNA-LNPs) and in the absence of Triton X (to measure the unencapsulated mRNA concentration). Finally, mRNA encapsulation efficiency (EE%) was calculated.

### 2.5. In Vitro Expression

CHH-1 cells, originally established from heart tissue from chum salmon (*Oncorhynchus keta*) [35], were cultured in Leibovitz’s L-15 medium (ThermoFisherScientific, Waltham, MA, USA), supplemented with 10% fetal bovine serum and 50 mg/mL gentamycin at 20 °C. For experiments, cells were seeded in 12-well plates, and when 90% cell confluency was reached, 500 ng LNP-encapsulated mRNA encoding EGFP was transfected per well, corresponding to 140 ng of encapsulated mRNA/cm^2^. Expression of EGFP was monitored during a period of 11 days, using an EVOS Microscope M5000 Imaging System fluorescence microscope (ThermoFisherScientific). Negative controls were either cells transfected with mRNA-LNPs encoding Fluc or cells included with 10 mM TRIS-HCl with 10% sucrose. Images were prepared for publication using brightness and contrast adjustments with ImageJ, with the same settings applied to all images.

### 2.6. In Vitro Cytotoxicity of mRNA-LNPs

CHH-1 cells were seeded in 24-well plates at approximately 50,000 cells per well. Then, 24 h after cell seeding, cells were transfected with 285 or 570 ng EGFP-encoding mRNA encapsulated in LNPs, corresponding to 150 or 300 ng encapsulated mRNA/cm^2^, respectively. Cytotoxicity was monitored using the CellTiter 96^®^ AQueous One Solution Cell Proliferation Assay (MTS, Promega) 1, 3, and 5 days after transfection. The medium was removed, washed with PBS, and replaced with fresh medium before the CellTiter 96^®^ AQueous One Solution was added, and then it was incubated for 4 h at 20 °C. Finally, the amount of formazan, which is produced when metabolically active cells reduce the MTS tetrazolium salt, was recorded at 490 nm with a reference wavelength of 650 nm using a TECAN SPARK spectrophotometer (TECAN, Männedorf, Switzerland).

### 2.7. Injection of mRNA-LNPs in Salmon

This in vivo study was conducted at the VESO Aqualab research facility (Vikan, Norway). The experiments had been approved by the Norwegian Food Safety Authority according to the European Union Directive 2010/63/EU (permit number FOTS ID 22074). The fish were unvaccinated Atlantic salmon with an average body weight of 40 g. Fish were seronegative for specific antibodies against *Vibrio salmonicida*, *Vibrio anguillarum O1 and O2a*, *Vibrio ordalii*, *Aeromonas salmonicida subsp. salmonicida*, *Moritella viscosa*, and *Yersinia ruckeri* and had tested negative by qPCR for infectious pancreatic necrosis virus, ISAV, salmon pancreas disease virus, piscine orthoreovirus, and piscine myocarditis virus before the initiation of the study. The fish were kept under a 12:12 light–dark regime in 12 °C fresh water. The fish were anesthetized by immersion in benzocaine chloride (5 ± 2 min, 0.5 g/10 L water) prior to handling, and euthanized using a lethal dose of benzocaine chloride (1 g/5 L water). The fish were observed at least once per day. No fish died during the trial. Four salmon fish were injected intramuscularly into white muscle tissue at a fixed injection site near the dorsal fin with 100 µL of the mRNA-LNP solution containing 1 µg of EGFP-encoding mRNA. Two negative controls were used: one injected with 1 µg of Fluc-encoding mRNA-LNPs, and one that did not receive any injection. Muscle tissue samples from the injection site were collected 7 days post injection (dpi), fixed in formalin for 24 h, and stored in ethanol at 4 °C until paraffin embedding.

### 2.8. Immunohistochemistry

Immunohistochemistry was performed with an MACH 1 Universal HRP-Polymer Detection kit (Biocare Medical, Pacheco, CA, USA). Tissue from mice expressing EGFP was included for method validation and was kindly gifted by Guttorm Haraldsen (University of Oslo and Oslo University Hospital, Oslo, Norway). Sections were dewaxed and subjected to heat-mediated antigen retrieval in DIVA solution at 100 °C for 10 min. Endogenous peroxidase was blocked by adding Peroxidazed 1 and incubated for 10 min at room temperature (RT). Furthermore, blocking of unspecific binding was performed by incubating the sections with Background Sniper for 10 min at RT. Anti-GFP antibody (A-6455, Invitrogen, ThermoFisherScientific) was diluted 1:1000 in Da Vinci Diluent, and the tissue sections were incubated with the antibody at RT for 1 h in a humidified chamber. The tissue sections were then incubated with MACH 1 Universal HRP-Polymer for 30 min at RT, and 3,3′-diaminobenzidine (DAB) was used to visualize the immunoreaction. Tissue sections were counterstained with hematoxylin before dehydration in 100% ethanol, clearing in xylene, and mounting. A Nikon Eclipse Ni-U microscope (Nikon, Tokyo, Japan) was used to visualize the muscle tissue samples.

## 3. Results

### 3.1. Production of mRNAs and LNP Encapsulation

**Figure 2:**
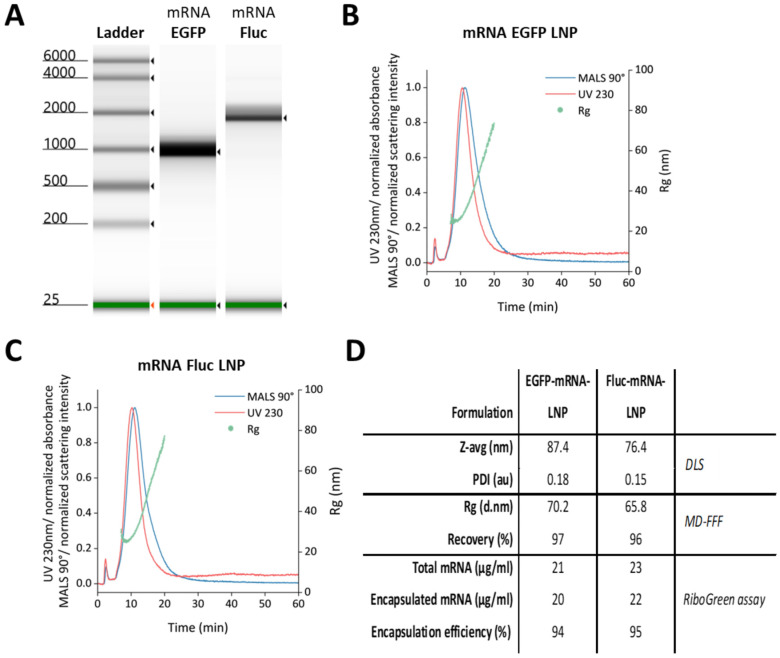
Generation of EGFP and Fluc mRNA, and LNP encapsulation. (**A**) Evaluation of mRNA quality and size after IVT of EGFP and Fluc mRNAs with the use of a Tapestation 4200 instrument. (**B**,**C**) MD-FFF fractograms for the 2 mRNA-LNP formulations. (**D**) Summary of mRNA-LNP characteristics obtained with dynamic light scattering (DLS), multi-detector field flow fractionation (MD-FFF), and the RiboGreen assay.

mRNAs were transcribed in vitro from plasmids encoding EGFP or Fluc, and their size and integrity was evaluated using capillary gel electrophoresis. We confirmed the size of the EGFP construct to be about 1000 nt and that of the Fluc mRNA to be 1800 nt (Figure 2A). The constructs were encapsulated in LNPs with a lipid composition very similar to that of the Moderna COVID-19 vaccine. The obtained mRNA-LNPs had a z-average diameter below 90 nm and a PDI below 0.2 as measured by DLS, indicating successful formulation. MD-FFF analysis confirmed a high quality of the formulations. The sharp single peaks in the elution profiles (fractograms; Figure 2B,C) demonstrated a well-defined, single population of mRNA-LNPs. The high recovery (>96%) indicated that nearly all material in the formulation was present as mRNA-LNPs. Similarly, the high encapsulation efficiency (>94%), as measured with the RiboGreen assay, demonstrated efficient mRNA encapsulation. See Figure 2D for a summary of the mRNA-LNPs’ characteristics.

### 3.2. In Vitro Expression of mRNA-LNPs in CHH-1 Cells

**Figure 3:**
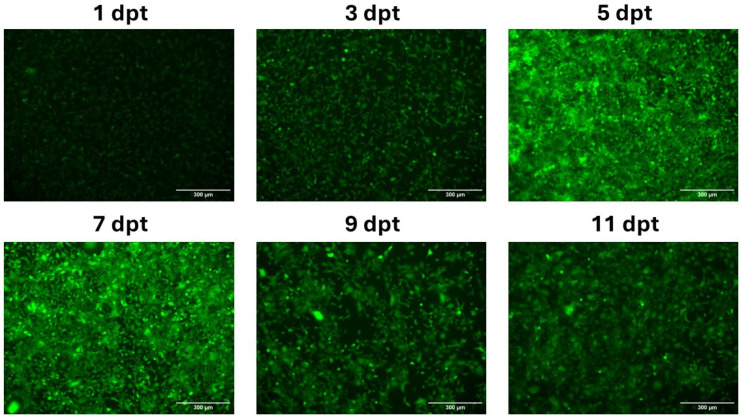
Transfection of mRNA-LNPs encoding EGFP in CHH-1 cells: 500 ng of mRNA-LNP encoding EGFP was transfected in CHH-1 cells and protein expression was monitored by fluorescence microscopy analysis over 11 days.

CHH-1 cells were transfected with mRNA-LNPs encoding EGFP to evaluate the capability of LNPs to transport mRNAs transcribed in vitro and to confirm that the N1-methyl-pseuoduridine-modified mRNA induced protein expression in salmonid cells. The CHH-1 cells were monitored over 11 days post transfection (dpt) and revealed protein expression as early as 1 dpt and 3 dpt, although with a weak EGFP intensity. The expression of EGFP peaked between 5 dpt and 7 dpt, and fluorescence microscopy indicated that most cells expressed EGFP. The production of EGFP started to decline at 9 dpt, which continued until 11 dpt (Figure 3).

### 3.3. Evaluation of mRNA-LNP Cytotoxicity

**Figure 4:**
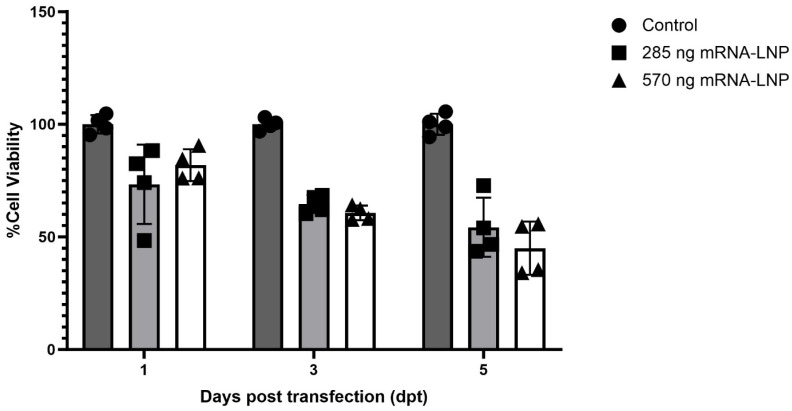
Evaluation of cell cytotoxicity of mRNA-LNPs compared to non-transfected control cells. Cell viability was monitored with an MTS assay at 1, 3, and 5 dpt in non-transfected control CHH-1 cells or CHH-1 cells treated with 285 ng or 570 ng of mRNA-LNPs.

An MTS assay was used to assess the cytotoxic effects when transfecting 285 or 570 ng of EGFP-encoding mRNA-LNPs in CHH-1 cells. The assay revealed 73% and 82% viability at 1 dpt in cells treated with mRNA-LNPs containing 285 ng or 570 ng mRNA, respectively, when compared to non-transfected control cells. The viability decreased for both mRNA-LNP concentrations at 3 dpt, with cells transfected with 285 ng mRNA-LNPs showing 64% viability compared to the control cells, and cells transfected with 570 ng mRNA-LNPs showing 60% viability. A further decrease in viability in the mRNA-LNP-transfected cells was observed at 5 dpt, where cells transfected with 285 ng or 570 ng of mRNA-LNPs showed 54% or 45% viability compared to control cells, respectively (Figure 4).

### 3.4. In Vivo Protein Expression

**Figure 5:**
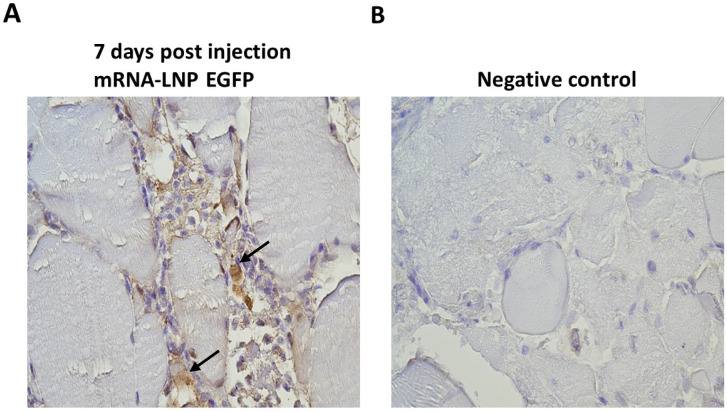
Immunohistochemical detection of EGFP expression following mRNA-LNP injection. Atlantic salmon were injected with 1 µg of mRNA-LNP encoding EGFP or Fluc, and tissue samples were collected at 7 dpi. Formalin-fixed paraffin-embedded tissue sections were stained with an antibody detecting GFP. (**A**) Muscle tissue injected with mRNA-LNPs encoding EGFP showed a focal inflammatory response in white skeletal musculature, where some leukocytes exhibited positive signal (brown areas, highlighted with arrows), while others did not. (**B**) The negative control tissue was sampled from salmon injected with mRNA-LNPs encoding Fluc and showed no positive signal.

A 100 µL amount of mRNA-LNP suspensions containing 1 µg of mRNA encoding EGFP or Fluc was injected intramuscularly into the salmon, and muscle tissue samples were collected at 7 dpi based on the peak of the protein expression as assessed in vitro. Immunohistochemistry was performed to assess EGFP expression. The muscle tissues collected from the site of injection exhibited a signal after staining with anti-GFP antibodies, confirming the presence of EGFP (Figure 5A). This signal was observed in cells infiltrating the space between the muscle cells. A focal immuno-positive inflammatory response was found in the white skeletal musculature. Some leukocytes exhibited a distinct EGFP signal, while others showed no signal. The myocytes appeared intact and showed no signal for EGFP. Taken together, this demonstrated the LNP technology’s capability to transport mRNAs into salmon cells effectively in vivo.

## 4. Discussion

To our knowledge, no studies have investigated the use of mRNAs delivered by LNPs as a tool for generating antigens in salmonid fish. The high need for improved vaccines in the salmon farming industry combined with the demonstrated and increasing potential of mRNA-LNP vaccines motivated us to assess whether this technology can be translated to salmon. Our study aimed to determine the effectiveness of LNPs in transfecting mRNA into salmon cells and inducing the expression of corresponding proteins. Our findings indicate that this technology may be adopted in vaccine strategies against piscine pathogens. While previous studies have shown the ability of mRNA-LNP technology to successfully express proteins in zebrafish [36], important biological and environmental disparities exist between zebrafish and salmonids. For example, these species have adapted to very different temperatures, approximately 25–30 °C for zebrafish and 5–15 °C for salmonids, and variations in temperature might influence the functionality of LNPs [37].

In the present study, mRNAs were synthesized by IVT from a DNA plasmid containing UTRs from the ISAV HE glycoprotein sequence and the coding sequence for EGFP. The DNA plasmid also included a 120-base-long poly(A) tail, which ensures the reproducibility of IVT mRNA with a fixed poly(A) length, as opposed to an enzymatic post-transcriptional addition of the poly(A) tail [18]. Additionally, the mRNA transcript was modified with N1-methyl-pseudouridine to enhance protein translation and mRNA stability [16], suppress innate immune responses [38,39,40], and reduce the formation of double-stranded RNA (ds RNA) contaminants, as ds RNA is a powerful inducer of the innate immune response [41]. LNPs were engineered to enhance the in vivo transfection efficiency of mRNA, increasing subsequent protein expression. Different modifications have been implemented to refine LNP characteristics, including the utilization of ionizable cationic lipids [42]. Herein, we employed LNPs with ionizable cationic lipids to evaluate their ability to deliver mRNAs encoding EGFP into salmonid cells.

First, to determine the ability of LNPs to effectively transport mRNA into salmonid cells, mRNA-LNPs encoding EGFP were transfected into CHH-1 cells. EGFP expression was observed as early as 1 dpt, and the intensity of EGFP fluorescence activity steadily increased until 7 dpt. Notably, EGFP activity persisted even after 11 dpt, indicating the sustained protein expression achieved through mRNA-LNP transfection. The formulation of the LNPs we used was originally developed for use in mammals; thus, their functionality is tailored to this specific biological environment, maintained at 37 °C. Therefore, it is important to establish that these mRNA-LNPs are internalized in salmonid cells and can induce protein translation from the encapsulated mRNA. In addition, the N1-pseudouridine modification raised some questions regarding functionality in salmonid cells. The presence of pseudouridine synthase has been detected in Chinook salmon (*Oncorhynchus tshawytscha*) [43], which indicates the natural appearance of pseudouridine in the RNAs of salmonid fish. The production of functional EGFP in CHH-1 cells showed that N1-methylated pseudouridine-modified mRNA can instruct the correct translation of functional proteins in salmonid cells.

LNPs have previously been shown to possess adjuvant activity, which is important for achieving a robust adaptive immune response to vaccination [44].

The viability assay revealed a decrease in cellular metabolic activity in cells transfected with mRNA-LNPs as early as 1 dpt, compared to non-transfected control cells, and this was more pronounced at 3 and 5 dpt. The decreased cellular metabolic activity indicated that these mRNA-LNPs have potential to cause cytotoxicity in salmon cells, at least in vitro. This was also observed microscopically, where a significant decrease in cell density occurred after washing. This indicated decreased cell viability and a decreased ability of the cells to stick to the well surface, compared to the control cells where the density remained the same after washing. It should be kept in mind that the cytotoxic effect of these mRNA-LNPs might differ between different cell lines [45]. The observed cytotoxicity could be attributed to the lipid component, the mRNA component, or a combination of both. The cytotoxicity of LNPs optimized for mammalian cells at 37 °C could be different in fish cells adapted to temperatures below 20 °C, due to, for example, differences in plasma membrane fluidity.

To assess the in vivo performance of these mRNA-LNPs, salmon were injected intramuscularly with mRNA-LNPs encoding EGFP, and muscle tissue samples were harvested at 7 dpi, which is within the time frame of high in vitro protein expression in CHH-1 cells. The muscle tissue samples were subjected to IHC analysis, which visualized the presence of EGFP. Importantly, for the in vivo analysis, the salmon were kept at their natural temperature, ensuring that the experiment was conducted in a biologically relevant environment. The presence of EGFP indicated that LNPs can be taken up by cells in salmon muscle tissue and induce correct translation of the encapsulated mRNA. EGFP expression was mostly observed in infiltrating phagocytes in the space between the muscle cells, while there was no expression in the muscle fiber that could be visually observed. This is in accordance with the localization of protein expression observed after intramuscular administration of mRNA-LNPs in rodents [46]. The accumulation of phagocytes indicates that the LNPs were recognized as foreign material and induced an inflammatory response. The phagocytic cells recognize and internalize foreign material such as the administered mRNA-LNPs, and their response demonstrates the functionality of the tested LNPs beyond their original application in mammals, suggesting their potential as efficient delivery vehicles for mRNA in salmonid fish.

It should be noted that the current work was performed with an LNP composition developed for use in humans. An optimization of the delivery system according to the physiology and immunology of salmon, and a concomitant quantitative comparison of the efficacy of different LNPs, could be interesting for further development.

## 5. Conclusions

This study demonstrates the successful use of LNPs for mRNA delivery in salmonids, ensuring protein expression in salmonid cell cultures in vitro and in salmonid fish in vivo. The production of EGFP in response to mRNA-LNP injection in salmon suggests that this vaccine technology has promise for inducing the production of immunizing antigens to counteract pathogens infecting salmonids and potentially other fish species.

## Figures and Tables

**Figure A1:**
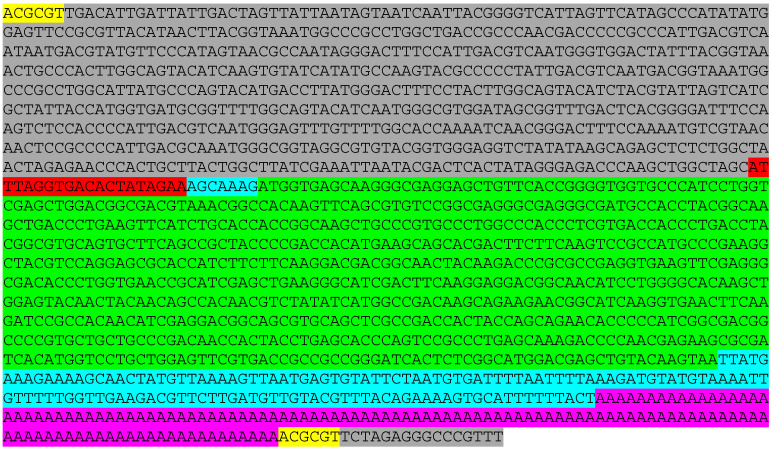
DNA sequence used for in vitro transcription of EGFP-encoding mRNA. The different sections are highlighted in color codes: MluI restriction sites; pVax1 Vector; UTRs; SP6 promoter; EGFP coding sequence; Poly(A).

## Data Availability

The raw data supporting the conclusions of this article will be made available by the authors, without undue reservation.
